# Supplementary material for: 222-Nanometer Far-UVC Exposure Results in DNA Damage and Transcriptional Changes to Mammalian Cells
Source: Int J Mol Sci. 2022 Aug 14;23(16):9112. doi: 10.3390/ijms23169112 (PMC9408858; doi:10.3390/ijms23169112)
Supplement: Supplementary file 1 [file ijms-23-09112-s001.zip › 20220813_supp.pdf]

Supplemental Tables

Table S1. Key Resources Table.

REAGENT or RESOURCE	SOURCE	IDENTIFIER
Antibodies		
P44/42 MAPK (Erk1/2)	Cell Signaling Technology	Cat: #9102, 1:1000
Phospho-p44/42 MAPK (Erk1/2) (Thr202/Tyr204)	Cell Signaling Technology	Cat: #9101, 1:1000
P38 MAPK (D13E1) XP(R)	Cell Signaling Technology	Cat: #8690, 1:1000
Phospho-p38 MAPK (Thr180/Tyr182) Antibody	Cell Signaling Technology	Cat: #9211, 1:1000
SAPK/JNK	Cell Signaling Technology	Cat: #9252, 1:1000
Phospho-SAPK/JNK (Thr183/Tyr185) Antibody	Cell Signaling Technology	Cat: #9251, 1:1000
GAPDH	Santa Cruz	sc-32233, 1:2000
Phospho-ATF-2(Thr71)	Cell Signaling Technology	Cat: #27934, 1:1000
Phospho-PERK(Thr981)	Cell Signaling Technology	Cat: #3179, 1:1000
Phospho-EIF2a (Ser51)	Cell Signaling Technology	Cat: #9721, 1:1000
EIF2a	Cell Signaling Technology	Cat: #9722, 1:1000
4-HNE	Abcam	Ab46545, 1:1000
Chemicals, peptides, and recombinant proteins		
DMEM:F12	ATCC	30-2006
RPMI-1640	ATCC	30-2011
Critical commercial assays		
Keratinocyte Growth Kit	ATCC	
Experimental models: Cell lines		
ARPE-19	ATCC	CCL-2302
HEKA	ATCC	PCS-200-011
Oligonucleotides – Primers for QPCR		
CAGCACATGACGGAGGTTGT		P53 forward
TCATCCAAATACTCCACACGC		P53 reverse
CTCTCGTCAGGCTTGAGTTTG		Rb1 forward
GACATCTCATCTAGGTCAACTGC		Rb1 reverse
ACGCTATGAGACCTCACTGAA		E2f1 forward

TCCTGGGTCAACCCCTCAAG		E2f1 reverse
CGTCCCTGAGTTCCCAACC		E2f2 forward
GCGAAGTGTCATACCGAGTCTT		E2f2 reverse
AGAAAGCGGTCATCAGTACCT		E2f3 forward
TGGACTTCGTAGTGCAGCTCT		E2f3 reverse
GAACGTCGAAAAGAAAAGTCTCG		Hif1a forward
CCTTATCAAGATGCGAACTCACA		Hif1a reverse
ATGGCTACCTCTCGATATGAGC		CDK4 forward
CATTGGGGACTCTCACACTCT		CDK4 reverse
AGACAGCCACTCACCTCTTCAG		IL6 forward
TTCTGCCAGTGCCTCTTTGCTG		IL6 reverse
TCTGCCAACTACTCCCAGGT		NRF2 forward
GGGAATGTCTGCGCCAAAAG		NRF2 reverse
TCCACAGCACTGGTCTTGAG		BMP4 forward
GGGATGTTCTCCAGATGTTCTT		BMP4 reverse
TGCCCTCAAGATGCACATCCGA		SNAI1 forward
GGGACAGGAGAAGGGCTTCTC		SNAI1 reverse
AGAAAATCTGGCACCACACC		ACTB forward
TAGCACAGCCTGGATAGCAA		ACTB reverse
Software and algorithms		
ImageJ	Schneider et al., 2012	https://imagej.nih.gov/ij/
GraphPad Prism Sotware 8.4.3	GraphPad Inc.	
Quantstudio 5 software	Applied Biosystems	
Other		
222-nm far UVC lamp	Ushio	N.A.
254-nm UVC mercury lamp	Sankyo Denki	G8T5
277-nm UVC LED	Lextar	PU35CM1
Spectroradiometer	GL Optic	GL Spectis 4.0

Supplemental Figures

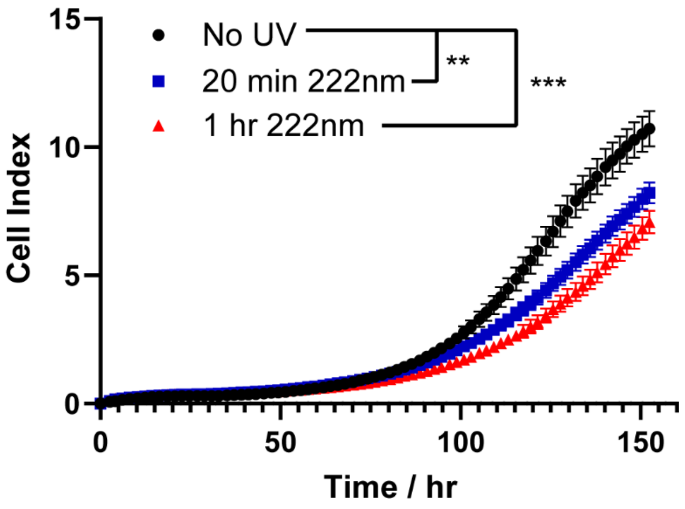

Figure S1: Dose-dependence curves of ARPE-19 cells subjected to no UV, 20 minutes of 222-nm illumination and 1 hour of 222-nm illumination. (c) Dynamic monitoring of cell numbers through xCelligence platform. Values are reported as mean +/- SD from n = 8 replicates. Where applicable, Student's t-test is performed and significance is represented as ** $p < 0.01$ and *** $p < 0.001$.

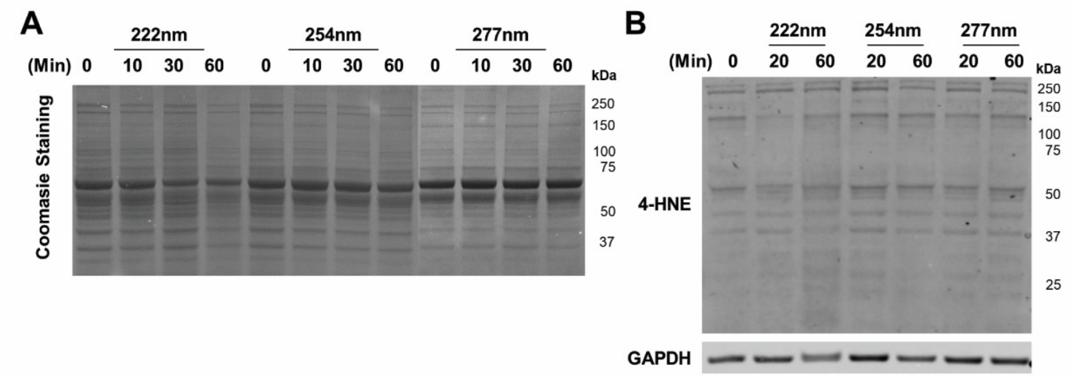

Figure S2: Different UVC wavelength result in varied levels of protein and lipid damage. **(A)** Coomasie staining of protein lysates of ARPE-19 cells subject to 0, 10, 30 and 60 minutes of respective UVC wavelength. **(B)** Western blot analysis of 4-HNE adducts was performed on protein lysates of ARPE-19 cells subject to 0, 20 and 60 minutes of respective UVC wavelength.

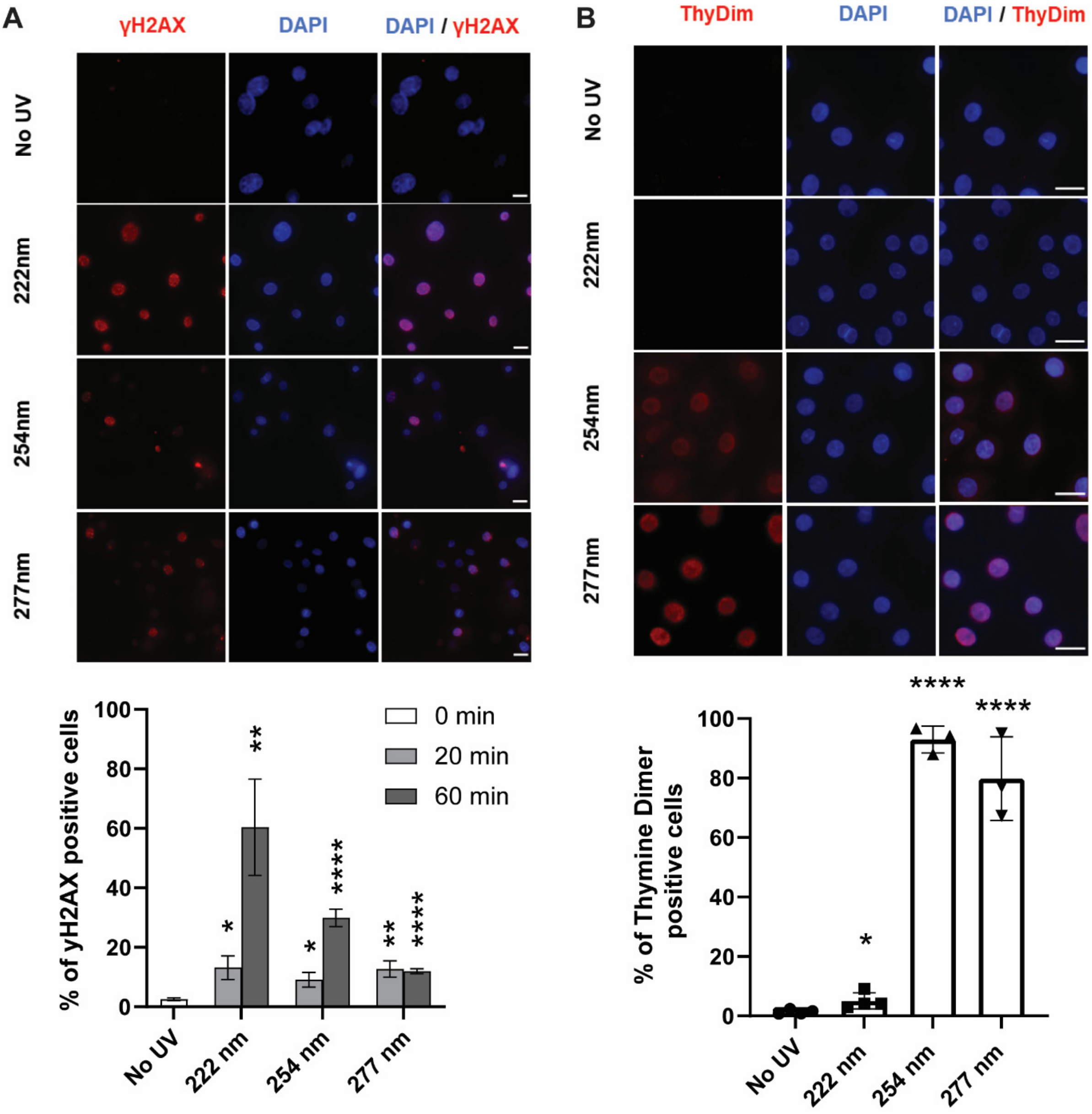

Figure S3: (A) γH2AX staining in HEK-A cells upon 60 minutes of respective UVC irradiation. Scale bars = 50 μm. Quantification of the extent of γH2AX activation in the HEK-A cells upon 0, 20 and 60 minutes of UVC illumination. Values are reported as mean +/- SD from n = 3 experiments. **(B)** Thymine dimer staining in HEK-A cells upon 60 minutes of respective UVC irradiation. Scale bars = 20 μm. Quantification of the extent of thymine dimer formation in the HEK-A cells upon 60 minutes of UVC illumination. Values are reported as mean +/- SD from n = 3 experiments. Where applicable, Student's t-test is performed and significance is represented as * $p < 0.05$, ** $p < 0.01$, and **** $p < 0.0001$.

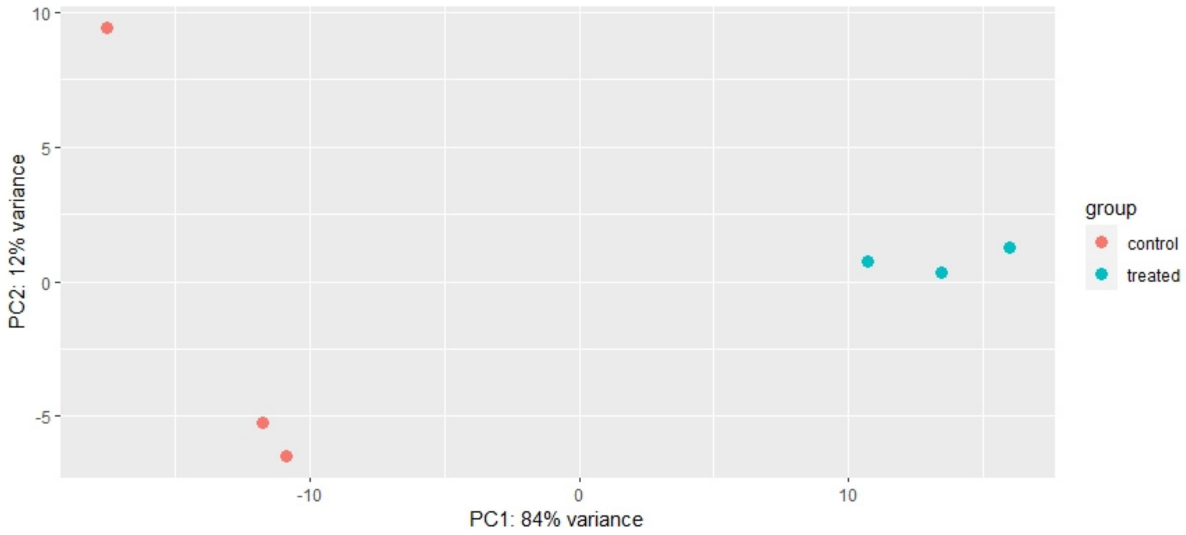

Figure S4: Principal component analysis of RNA sequencing results.

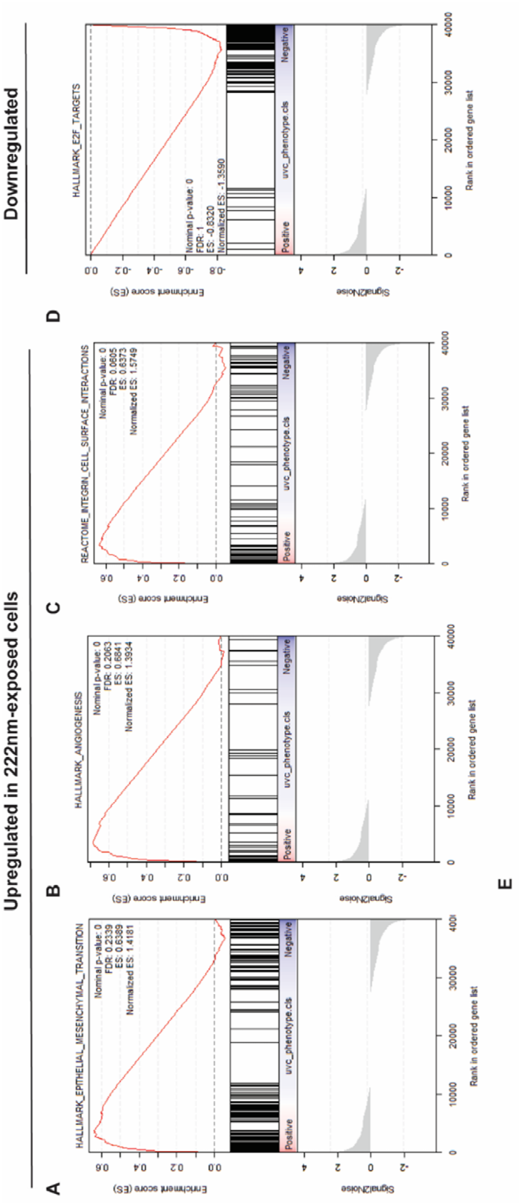

Figure S5: Further GSEA analysis including upregulated pathways in 222-nm lit cells in (A) Epithelial-mesenchymal transition, **(B)** Angiogenesis, **(C)** Integrin-cell surface interaction and

Supplemental Files

File S1: Top differentially expressed genes between 222-nm lit versus unlit cells.

File S2: Top regulator pathways as predicted by IPA analysis.

File S3: Top predicted changes in upstream regulators from IPA analysis.
